# Caffeine Consumption in a Group of Adolescents from South East Poland—A Cross Sectional Study

**DOI:** 10.3390/nu13062084

**Published:** 2021-06-18

**Authors:** Ewa Błaszczyk-Bębenek, Paweł Jagielski, Małgorzata Schlegel-Zawadzka

**Affiliations:** 1 Department of Nutrition and Drug Research, Institute of Public Health, Faculty of Health Sciences, Jagiellonian University Medical College, 31-066 Krakow, Poland; paweljan.jagielski@uj.edu.pl; 2 Collegium Masoviense High School of Health Sciences, 96-300 Zyrardów, Poland; m.schlegelzawadzka@gmail.com

**Keywords:** caffeine, caffeine intake, sources of caffeine, adolescents, risk assessment

## Abstract

Caffeine is the most common psychoactive substance available to adults, as well as to children and adolescents. The safety of its use in younger age groups requires further research. The aim of this study was to evaluate caffeine intake, to identify products and drinks that are the main sources of caffeine intake in the diet of the subjects and the risk of excessive caffeine intake with the diet of adolescents, stratified by gender. A cross-sectional study was conducted among 508 adolescents aged 16–18 years from southern Poland. Black tea, cola-based soft drinks and milk chocolate were the most frequently consumed products containing caffeine in the diet of the examined persons. The average caffeine intake was 95.54 mg/day (1.54 mg/kg b.w.). In 12.2% of the subjects the dose of 3 mg/kg b.w./day was exceeded, and in over 41.3% the dose causing sleep disorders was exceeded. The dose causing anxiety was also exceeded in 18.1% of the respondents, significantly more often in girls than boys (*p* = 0.0487).

## 1. Introduction

Caffeine is one of the best-described stimulants in the world and the products that are its source in the diet are equally popular among adults and younger age groups [1]. Recent studies have confirmed the safety of coffee consumption by adults, consumed in the form of hot drinks [1,2]. Many studies have shown that coffee may, among others, reduce the risk of type 2 diabetes and liver cancer, uterine or prostate cancer, and basal cell carcinoma. It was also shown to reduce the risk of neurological and cardiovascular diseases [3]. Drinking coffee in the amount of 3–4 cups a day was also associated with reduced mortality in a group of women and men in the HAPIEE study [4]. However, the safety of caffeine, which is the main stimulant present in coffee, depends on individual characteristics, such as body weight, gender, physiological condition or the dose consumed [1,5]. Caffeine occurs naturally in coffee, tea or chocolate [6]. Synthetic caffeine is found in many products that are used to eliminate sleepiness and provide psychophysical energy [7]. The sources of caffeine in the diet do not only include drinks such as coffee or tea, drugs and dietary supplements, but also energy drinks or chocolate, popular among the youth [6,7]. The safety of caffeine used by adolescents is constantly studied, and establishing its safe level of consumption in children and adolescents is difficult to achieve [1,5,7]. Some recent reports on caffeine safety presented by the European Food Safety Authority (EFSA) [5] are slightly more restrictive in relation to the 2003 report published by Nawrot et al. [8]. A new study by Temple et al. revealed that caffeine consumption was associated with risky behaviors undertaken by children and adolescents, regardless of gender [9], as well as the increased consumption of sweet soft drinks among girls [10]. Caffeine consumption by adolescents is also associated with sleep disorders, which may lead to learning and concentration difficulties, or even an increased risk of obesity [11]. In Kim’s study, higher caffeine intake was also associated with lower nutritional knowledge and knowledge about the effects of caffeine on bones and sleep disorders [12]. According to the NHANES study, children who watched TV more than two hours a day were characterized by a significantly higher caffeine intake [13]. The most common side effects of the consumption of caffeinated products or beverages include insomnia, frequent urination, excitement or anxiety, and headaches [14,15]. Therefore, it is necessary to conduct further research into the assessment of caffeine consumption and dietary intake in children and adolescents.

## 2. Materials and Methods

The study was designed as a cross-sectional survey, carried out in 2014–2015 and covering data of 508 secondary school students aged 16–18. The study included 246 boys and 262 girls from two environments—rural and urban from the southern regions of Poland (Subcarpathian voivodeships). The research was conducted among students from schools where the school principals had given their prior consent to conduct the study. All persons between the ages of 16–18 who gave their written consent to participate in the study (in the case of people over 16 years old, the informed consent was also signed by their guardians) and in whom it was possible to take anthropometric measurements participated in the study. The study was conducted in accordance with the Declaration of Helsinki for medical research [16] and with the positive approval of the Jagiellonian University Bioethics Commission (KBET/62/B/2013). This paper was prepared according to the Strengthening the Reporting of Observational Studies in Nutritional Epidemiology (STROBE-nut) checklist [17].

The research tool for the assessment of caffeine intake with diet was a self-reported questionnaire containing questions about the frequency and amount of caffeine-containing products consumed in the diet. The ADOS-Ca questionnaire, used for calcium intake evaluation, was the source of frequency ranges proposed for the assessment of the average caffeine intake per day [18]. The questionnaire had been validated in earlier studies concerning the assessment of caffeine participation in the diet of junior high school students [19]. The scale included eight possible answers from which the respondents chose one (never, less frequently than once a week, 1–2 times a week, 3–4 times a week, 5–6 times a week, once a day, 2 times a day, 3 times a day). The average caffeine intake was determined (more than the basic dose, basic dose and half the basic dose) by choosing one of three possible dose sizes. Depending on the product, the basic serving was one cup (150 mL, ground black coffee, instant coffee, 3-in-1 coffee, 2-in-1 coffee, cappuccino), a cup/glass (250 mL, black tea, green tea, cocoa, cola-based soft drinks and energy drinks) and a bar of chocolate (100 g for milk chocolate, bitter chocolate). As regards the quantitative assessment of caffeine consumption by the examined adolescents, we used the average caffeine content in a portion of a product according to the size indicated in the survey. The mean values taking into account brewing time and the size of the portion to prepare the infusion (refers to beverages) were indicated basing on the studies in which such values had been determined [17,20,21]. Health risk assessment of caffeine intake with diet was based on the percentage of individuals with an intake exceeding the accepted doses considered safe at the usual EFSA intake of 3 mg/kg b.w. [5]. Specific adverse effects causing sleep disorders (>1.4 mg/kg b.w.), and anxiety (>2.5 mg/kg b.w.) were assessed according to the NNT (Nordic Working Group on Food Toxicology and Risk Evaluation) [22].

The analysis of quantitative data on caffeine intake with diet was performed with Microsoft Office Excel, with which the average caffeine dietary intake was calculated. The comparison of the collected quantitative data between the distinguished groups, by place of residence and gender, was performed with the non-parametric Mann-Whitney U test and Chi^2^. The level of significance was assumed at α = 0.05. Statistical calculations were performed with STATISTICA PL 13.0 (JU license, TIBCO Software Inc., Palo Alto, CA, USA).

The interpretation of BMI value was made using centile charts developed in Polish OLAF research [23]. Abdominal obesity was identified using the Waist to Height Ratio (WHtR) indicator with the constant cut-off value for both genders of ≥0.5 [24].

## 3. Results

A total of 599 students took part in the study, but the analysis included the results obtained from 508 respondents who correctly completed the questionnaire on the frequency of consumption of caffeinated products. As regards the study group, 34.3% of the participants drunk one cup of cocoa (250 mL) and 29.1% drunk a cup of tea daily. One cup of cola-type beverages was drunk by 27.2% of the participants and nearly 21.5% of them drank energy drinks. Half a bar of milk chocolate or bitter chocolate (50 g) was eaten by 41.5% and 24% of adolescents, respectively.

The characteristics of the diet and nutritional status of the studied group with respect to the gender of the studied persons are presented in Table 1. The group included 48.4% of boys and 51.6% of girls. Over half of the respondents (59.3%) were urban residents and 40.7% inhabited the rural areas of southern Poland. The average age of the respondents was 17.11 ± 0.57 years and their body weight was 64.18 ± 12.63 kg.

### 3.1. Caffeine Intake

Caffeine was not present in the diet of all the subjects (minimum intake: 0.00 mg). The maximum daily intake of caffeine among the subjects was 665.1 mg (10.36 mg/kg b.w./day). The average daily caffeine intake in the studied group consumed with drinks and other products was estimated at 98.54 mg/day. From the perspective of body weight it translated into 1.54 mg/kg b.w./day. There were no differences in gender groups in caffeine intake during the day (*p* = 0.9534) or in the validated value of mg/kg of body weight (*p* = 0.0632; Table 2).

Furthermore, no statistically significant differences were found as regards caffeine intake with diet according to the place of residence (*p* = 0.3485). On average, adolescents from rural areas consumed 99.5 mg of caffeine per day (1.59 mg/kg b.w.). The respective value for city citizens was 89.8 mg (1.46 mg/kg b.w.) per day.

### 3.2. Sources of Caffeine in the Diet

**Figure 1:**
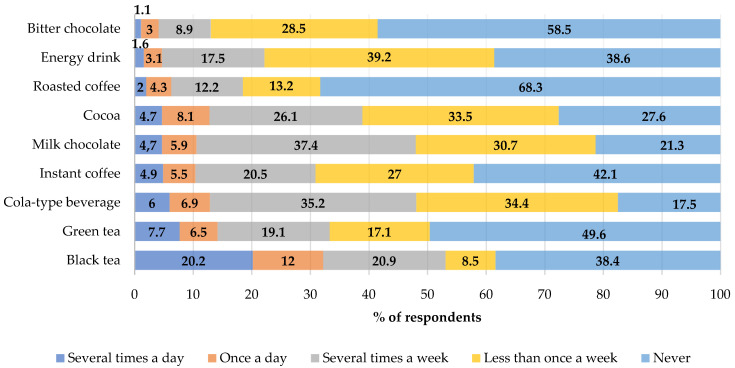
Frequency of the consumption of selected caffeine source products in the total study group (%).

Black tea was the most frequently chosen source of caffeine in the diet. It was drunk by 20.2% of the respondents daily. Bitter chocolate, which was eaten by only 1% of respondents daily, was the least frequently chosen product (Figure 1).

**Figure 2:**
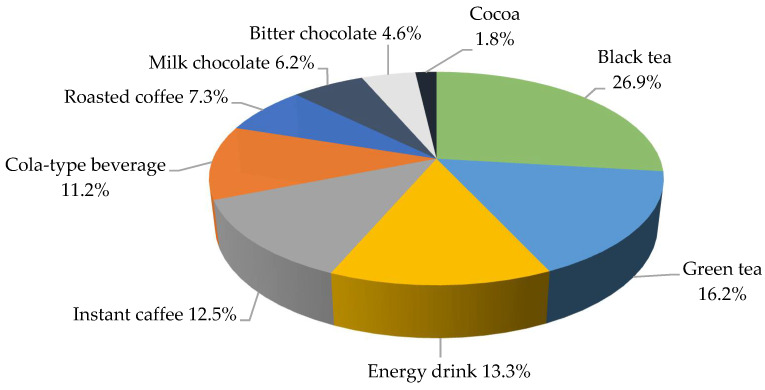
The share of the individual sources of caffeine in the diet of the study group during the day (%).

Boys drank significantly more often cola-based soft drinks (24.8% vs. 17.6%; *p* = 0.0401) and ate bitter chocolate than girls (31.7% vs. 25.6% *p* = 0.0219) per week. The weekly consumption of instant coffee was significantly more common in girls than in boys (16.8% vs. 8.1%; *p* < 0.0001). The highest amounts of caffeine in the daily diet of the adolescents were consumed with black tea (26.9%), followed by green tea, and energy drinks, as shown in Figure 2. No differences were demonstrated in the groups with respect to the place of residence and the main sources of caffeine in the diet (≥0.05).

The average caffeine intake was 25.72 ± 40.13 mg for black tea, 15.52 ± 35.55 mg for green tea and 12.73 ± 33.30 mg for energy drinks. As regards gender groups, boys consumed significantly higher doses of caffeine with black tea (28.07 ± 39.81 mg vs. 23.52 ± 40.38 mg; *p* = 0.0230) and cola-based soft drinks (11.47 ± 20.76 mg vs. 10.01 ± 10.01 mg; *p* = 0.0068) and girls with instant coffee (10.04 ± 229.31 mg vs. 13.78 ± 31.99 mg; *p* = 0.0003; Table 3).

### 3.3. Risk Assessment of Excessive Caffeine Intake

The dose of caffeine causing sleep disorders was exceeded in over 40% of the examined adolescents. Girls significantly more often consumed caffeine in amounts causing anxiety and fear (Table 4). At the same time, more than 12% of the subjects consumed caffeine at a dose above 3 mg per kg of body weight. Among the respondents, 4.3% consumed caffeine at a dose higher than 5 mg per kg of body weight with their diet.

## 4. Discussion

Analyses of the dietary intake of caffeine products in children and adolescents are currently being carried out in many countries. Nevertheless, attempts to estimate its contribution from all dietary sources are problematic due to the limited research in this area and methodological difficulties.

Black tea was the main source of caffeine for a group of adolescents in the present study. Black tea (46%) and coffee (26%) were also the main sources of caffeine in the examined teenagers from Warsaw aged 17.1 ± 0.9 years [25]. According to Guelinckx et al., hot beverages (coffee, tea and other hot beverages) constituted a high percentage of total fluid intake in the sample of children from Poland (34%). A similar percentage was identified in adolescents [26]. In a study among younger age groups (11–13 years of age) in Warsaw, the majority of participants drank cola-based soft drinks (89.7%). Energy drinks were consumed by 23.8% of the examined children [27], in our study it was respectively 82.5% for cola-based drinks and 61.4% for energy drinks. In a similar study, cola-based soft drinks and cocoa were the most frequently consumed beverages containing caffeine [19]. Cola-based soft drinks, as a source of caffeine, were used by 97% of school pupils from Kutno (town in Poland). A total of 51% of them drank coffee and 48% declared the consumption of energy drinks [28]. In contrast to our own research and that of other Polish authors, the nature of beverages that are the source of caffeine in the diet of young Americans is different. They most commonly reach for beverages containing caffeine: soda (33%), followed by tea (28%) and coffee (25%) [29].

Caffeine intake, from all sources, in our study was 95.5 mg per day. In a study among younger age groups (11–13 years of age) in Warsaw, the daily median caffeine consumption was 4 mg [27]. As regards the group of adolescents aged 15.4 ± 1.6 living in the Warsaw area, the lowest caffeine intake with diet (reported using the 3-day diary recording method) was higher than in our own and it was 152 mg per day [30]. The maximum caffeine intake in the cited study from Warsaw, assuming the highest caffeine content in products and the strongest infusions, was about 274 mg/day [30], being more than twice as high as in our own research. Another group of adolescents reported the average caffeine intake to be 196 mg per day, which was more than half as high as in our study. In this study, 38% of the students overdosed caffeine at the level of 3 mg/kg body weight. It was observed significantly more often in girls (48%) than in boys (28%, *p* = 0.032) [25]. As regards 15-year-olds, the average caffeine intake with diet was 141 mg/day, which was reported on the basis of a questionnaire on the frequency of the consumption of beverages containing caffeine [28]. According to the NHANES study conducted in a group children and adolescents a decrease in caffeine intake from 175 mg per day to 142 mg per day was observed over time. The average caffeine intake was the highest for soda (20 mg/day) followed by tea (17 mg/day) and coffee (15 mg/day). The average caffeine intake with diet in the study group of 14–19-year-olds was 61 mg per day [29] and was about 35 mg lower than in our study.

Energy drinks are a source of caffeine in the diet of young people. They are especially popular among European teenagers, where, according to the data provided by EFSA, the prevalence of caffeine consumption is 68%. In this European study, the consumption of energy drinks was declared by 73% of teenagers from Poland and the estimated caffeine intake was 171.44 mg/day [31]. The average caffeine content in 100 mL of an energy drink is about 30 mg [17,32]. The caffeine content per serving size (250 to 500 mL) is in the range of variation 35 to 160 mg [32]. The average caffeine content in the 330 mL energy drink is 80 mg [33] Energy drinks were responsible for 6 mg/day of caffeine intake in the diet of American teenagers [29]. As regards European countries, the average exposure to caffeine with energy drinks was as high as 23.5 mg/day. In Polish respondents it was 16.20 mg/day [31], and in the present study it was 12.73 mg/day. The popularity of energy drinks among teenagers was also confirmed by the results of a study by Martyn et al., where the share of caffeine from such drinks was the highest among all the drinks that are its source in the diet. An increase in the consumption of caffeine from such products was observed on Fridays [34].

Risk assessment of excessive caffeine intake was one element of our study. High caffeine consumption is associated with the risk of adverse symptoms for the health of young people. The present research showed that 12.2% of the respondents exceeded caffeine dose of 3 mg/kg mc/day. According to the literature, products containing caffeine were present in the diet of young people also from the Warsaw region. The consumption of products containing caffeine was over 300 mg/day in almost 40% of cases [30]. The dose of 3 mg/kg b.w was exceeded by 38% of adolescents from Wierzbicka et al. study [25].

Caffeine intake which caused sleep disorders was reported in as many as 41.3% of the examined teenagers. Higher than in own study, average intake of caffeine led to sleep disturbance in 77% of the subjects from Warsaw area [30]. Higher caffeine intake is associated with the average shorter sleeping time and time spent in bed [35]. A study conducted in a sample of the American population of more than 40,000 participants in various age groups (ages from 13 to 65+) showed that the distribution of daily caffeine intake from all sources was the highest in the morning and in the evening (34%) [34]. Insufficient amount and poor quality of sleep are a serious problem among adolescents [36]. Results from survey in a group high school students in a Polish city show that only 11.5% of adolescents get enough sleep [37]. The prevalence of sleep disorder among adolescents from Indonesia occurred in 22.8% participants [38]. Compared to the US population, the Youth Risk Behavior Survey found that as many as 72.7% of students reported an average of <8 h of sleep per school night [39]. Sleep disturbances in adolescents, caused inter alia, by the consumption of caffeine, can lead serious public health problems like an excess body weight [40,41] as shown in the study of a group of adolescents from selected cities of Upper Silesia, Poland [42].

The dose causing anxiety was exceeded in 18.1% of young people from presented study and was lower than in the cited previously group of adolescents where 46% reported anxiety [29]. Among the adolescents surveyed in the Polish HBSC study (Health Behaviour in School-age Children), 41% of boys and 48% of girls reported feeling anxious more than once a week. These results were higher than the average for European and North American respondents overall [43].

## 5. Conclusions

Despite the fact that caffeine consumption in the study group was relatively low, over 12% of the subjects exceeded the safe level of caffeine consumption. Even low caffeine consumption among teenagers, as shown in our study, can cause sleep disturbances. Sleep disorders can lead to excess body weight in adolescents, still an ongoing and very important public health problem.

## Figures and Tables

**Table 1:** Dietary habits and nutritional status of the study group according to gender.

Parameter	Total *n* = 508 %	Boys *n* = 246 %	Girls *n* = 262 %	*p*
Meals				
Breakfast	82.1	86.6	77.8	**0.0104**
Second breakfast	72.0	69.1	74.7	0.1826
Lunch	94.9	96.7	93.1	**0.0466**
Afternoon tea	41.4	40.2	42.5	0.6273
Dinner	84.2	92.7	76.2	**<0.0001**
Break Between Meals				
≤2 h	21.5	21.0	22.0	
3–4 h	62.4	62.9	61.9	0.9254
≥4 h	16.1	16.2	16.1	
Fast Food Consumption				
I do not eat	12.0	12.6	11.5	
Several times a month or less	67.9	63.4	72.0	0.0749
Once a week or more often	20.1	24.0	16.5	
BMI, Interpretation				
Underweight	15.4	10.1	20.3	**0.0068**
Normal	67.9	74.0	62.1	
Overweight	11.2	10.2	12.3	
Obese	5.5	5.7	5.4	
Abdominal obesity by WHtR	3.7	4.5	3.1	0.3999

*n*—number, *p—*level of statistical significance in the gender group, Chi^2^ test, BMI—Body Mass Index, WHtR—Waist-to-Height ratio, significant differences are marked in bold.

**Table 2:** Caffeine intake per day (mg) and per body weight (mg/kg b.w.).

Estimation of Caffeine Intake	Total *n* = 508 X (SD) Me Min-Max	Boys *n* = 246 X (SD) Me Min-Max	Girls *n* = 262 X (SD) Me Min-Max	*p*
Caffeine mg/day	98.54 (69.5)	96.21 (96.79)	94.91 (96.70)	0.9534
	69.5	71.70	66.40	
	0.00–665.1	0.00–535.13	0.00–665.1	
Caffeine mg/kg b.w./day	1.54 (1.6)	1.42 (1.50)	1.66 (1.70)	0.0632
	1.0	0.96	1.07	
	0.00–10.74	0.00–7.67	0.00–10.74	

*n*—number, X—mean, SD—standard deviation, Me—median, Min—minimum, Max*—*maximum, *p—*level of statistical significance in the gender group, U Mann-Whitney test, b.w.—body weight.

**Table 3:** Average caffeine intake in the gender groups (mg).

Sources	Total *n* = 508 X (SD)	Boys *n* = 246 X (SD)	Girls *n* = 262 X (SD)	*p*
Black tea	25.72 (40.13)	28.07 (39.81)	23.52 (40.38)	0.0230
Green tea	15.52 (35.55)	12.64 (31.16)	18.22 (39.10)	0.1363
Energy drink	12.73 (33.30)	14.89 (34.32)	10.70 (32.24)	0.1920
Instant caffee	11.97 (30.75)	10.04 (29.31)	13.78 (31.99)	0.0003
Cola-type beverage	10.72 (21.55)	11.47 (20.76)	10.01 (22.28)	0.0068
Roasted coffee	6.93 (25.96)	5.38 (21.79)	8.38 (29.32)	0.3518
Milk chocolate	5.92 (11.84)	6.64 (13.66)	5.25 (9.81)	0.6216
Bitter chocolate	4.40 (14.83)	5.63 (17.32)	3.25 (11.95)	0.0811
Cocoa	1.62 (3.48)	1.45 (3.39)	1.78 (3.57)	0.2375

*n*—number, X—mean, SD—standard deviation, *p—*level of statistical significance in the gender group, U Mann-Whitney test, significant differences are marked in bold.

**Table 4:** Risk assessment of caffeine intake considering selected cut-off points [%].

Cut-Off Points	Total *n* = 508	Boys *n* = 246	Girls *n* = 262	*p*
Caffeine >1.4 mg/kg b.w.	41.3	40.2	42.4	0.6273
Caffeine >2.5 mg/kg b.w.	18.1	14.5	21.4	0.0487
Caffeine >3.0 mg/kg b.w.	12.2	10.2	14.1	0.1731

*n*—number, *p*—level of statistical significance in the gender groups, Chi^2^ test, b.w.—body weight.

## Data Availability

Data sharing is not applicable to this article.
